# Anisotropic Tensile Characterisation of *Eucalyptus nitens* Timber above Its Fibre Saturation Point, and Its Application

**DOI:** 10.3390/polym14122390

**Published:** 2022-06-13

**Authors:** Xudong Chen, Yingyao Cheng, Andrew Chan, Damien Holloway, Gregory Nolan

**Affiliations:** 1 School of Civil Engineering, Suzhou University of Science and Technology, Suzhou 215011, China; chenxd@usts.edu.cn; 2 School of Engineering, College of Sciences and Engineering, University of Tasmania, Hobart, TAS 7001, Australia; andrew.chan@utas.edu.au (A.C.); damien.holloway@utas.edu.au (D.H.); 3 Australian Research Council, Centre for Forest Value, University of Tasmania, Hobart, TAS 7001, Australia; gregory.nolan@utas.edu.au; 4 Centre for Sustainable Architecture with Wood (CSAW), University of Tasmania, Launceston, TAS 7250, Australia

**Keywords:** eucalyptus, tension, wood anisotropy, failure envelope, fibre saturation point

## Abstract

Plantation-grown *Eucalyptus nitens* (*E. nitens*) has been grown predominantly for the pulp and paper industry. In this study, the suitability of *E. nitens* as a structural material is examined using static tensile tests in a universal testing machine. The anisotropic tensile behaviour of 240 *Eucalyptus nitens* small clear wood samples with a diversity of grain angles was examined in both dry and wet conditions. The samples had a highly anisotropic tensile characterisation in the context of both a low moisture content (*MC* = 12%) and a high moisture content (*MC* > its fibre saturation point, *FSP*). The results showed that, in a high moisture content condition, the wood showed a lower failure strength and more ductility at all grain angles than in a low moisture content condition. The underlying failure mechanism of *Eucalyptus* *nitens* timber in tension was determined in detail from the perspective of the microstructure of wood cellulose polymer composites. The mean tensile failure strengths perpendicular and parallel to the fibre direction were, respectively, 5.6 and 91.6 MPa for the low *MC* and 3.8 and 62.1 MPa for the high *MC* condition. This research provides a basis for using *E. nitens* as a potential structural tensile member. The moisture modification factors of Eucalyptus timber at a mean level are higher than those of the traditional construction material, *Pinus radiata*, implying that *E. nitens* is promising as a material to be used for tensile members in water saturated conditions.

## 1. Introduction

Timber remains the dominant building material in some countries because of its cost effectiveness and high energy efficiency compared to steel and concrete [1,2,3]. However, this has led to shortages in plantation softwood and hardwood from native forests [4,5]. Consequently, fast-growing plantation hardwoods such as *Eucalyptus nitens* have attracted increasing interest because of their sustainable supply [6,7,8]. To use this resource for structural applications, the mechanical properties of *E. nitens* must be known in order to establish design codes.

Much attention is currently paid to the safety reassessment of timber structures in the context of high moisture content (*MC*) [5,9,10]. Wood is a hygroscopic material, and its strength decreases significantly as the *MC* increases until reaching a moisture content above the fibre saturation point (*FSP*) [9,11]. The decrease in strength can be up to 50% for both hardwood and softwood species, for example, American Beech and *P. radiata* [11]. During the past decade, research has been undertaken on changes in strength below about 30% *MC* [12,13]. A few studies [9,14] have covered the whole wood *MC* range up to the *FSP*. These studies have mainly focused on coniferous species, and there is still a substantial lack of quantitative information on the properties of *E. nitens* timber with a high *MC*; most of the available studies characterise its flexural and compressive properties [5,10].

Previous studies have found that timber members can reach an average *MC* above 70% after exposure outdoors for 2 months [15]. Moisture contents of 30–70% are associated with decay, which causes degradation and a reduction in the durability of timber structures [16,17]. This reduction in the mechanical properties of wood results from the change in wood cellulose polymers, where hydrogen bonding between the cellulose polymers is reduced because of the bound water in the cell wall [18,19]. The risk of damage in *E. nitens* timber is therefore expected to increase at a high *MC*, and design values for engineered tensile *E. nitens* elements with a low and high *MC* are required. The design values for tensile members are usually evaluated parallel to the grain and determined by small clear specimen testing according to current standards [20].

There are many mechanical failure modes in wood: flexural failure, compressive and tensile failure both perpendicular and parallel to the grain, and shear failure [11,21]. In tensile and bending members such as trusses and beams, tensile failure is the paramount failure pattern [11,20,22]. Tensile strength, evaluated both parallel and perpendicular to the grain direction, is significantly affected by the loading direction and hence the anisotropic tensile characterisation needs to be considered when the load-to-grain angle changes [11]. This paper investigates the influence of a high *MC* on the anisotropic tensile characterisation of *E. nitens.* The underlying failure mechanism due to the cellular nature of *E. nitens* wood will be explored. Design values for *E. nitens* timber in tension parallel to the grain (TPA) were developed at both low and high *MC*s, with the goal of using plantation *E. nitens* in structural engineering as a new building material. As a comparison, the tensile strength for a grain angle of 0° at both low (12%) and high (>*FSP*) *MC*s of the commercial construction timber species, *P. radiata,* was also obtained.

The specific objectives of this study, for both low and high *MC* samples, are listed as follows:

- Obtain the stress–strain curves of *E. nitens* samples in tension at grain angles of 0°, 10°, 45°, and 90°;
- Obtain the design characteristic values for *E. nitens* timber in tension parallel to the grain;
- Determine the anisotropic tensile characterisation (failure strengths) of *E. nitens* wood samples at different load-to-grain angles;
- Investigate the impact of high *MC* and grain angle on the tensile behaviours of *E. nitens* wood.

## 2. Materials and Methods

For the first stage of studying this problem, the experiment focused on ideal (i.e., defect-free) examples of *E. nitens* wood. Stress–strain curves were obtained in tensile testing on small clear *P. radiata* and *E. nitens* samples, with both low and high *MC*s. This is because *P. radiata* is a traditional structural material and is widely used in the construction industry. In order to examine the suitability of *Eucalyptus nitens* as a structural material, the tensile performance of *Eucalyptus nitens* timber for a grain angle of 0° was compared with *P. radiata* as a comparison. In this paper, a low moisture content or dry condition means that the *MC* is roughly 12%, whilst a high moisture content or wet condition means that *MC* > *FSP* (30%) with an assumption that the *FSP* of *E. nitens* is 30% [23] and that of *P. radiata* is in the range of 21–30% [11]. The tensile failure strengths for both low and high *MC*s were determined from the stress–strain curves.

### 2.1. Sample Preparation

The two timber species (*P. radiata* and *E. nitens*) were examined, and a summary of the experiments is provided in Table 1. Two groups of samples were used in this study: Group I was employed for determining the design characteristic values, and Group II characterised the anisotropic tensile behaviours of *E. nitens* wood at both high and low *MC*s.

The samples of *P. radiata* were obtained from a local trader with dimensions of 600 × 50 × 10 mm, while the *E. nitens* samples were cut from *E. nitens* boards with dimensions of 1500 × 50 × 30 mm for Group I, and 700 × 600 × 50 mm for Group II. An industrial hardwood kiln was used to dry the boards [10]. The dry samples were stored indoors for three months where the relative humidity and temperature were 60% and 20–30 °C. The wet samples were prepared using the same method described in a previous study [5]; they were simply soaked in water for six weeks.

**Figure 1:**
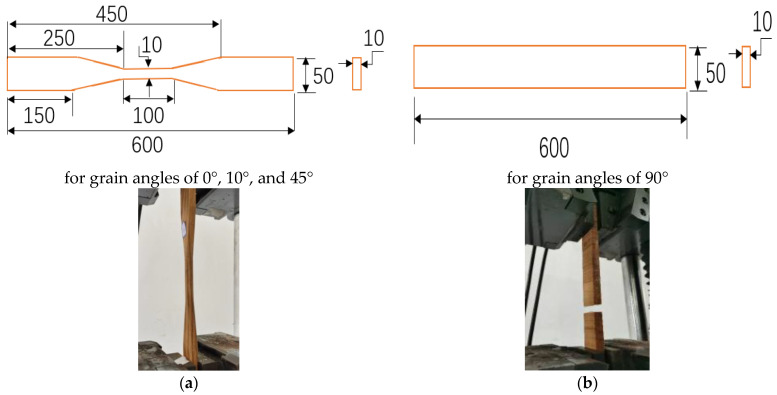
Dimensions of the samples: (**a**) sample dimension type for grain angles of 0°, 10°, or 45°; (**b**) sample dimension type for a grain angle of 90°.

For Group I, 160 small clear *P. radiata* samples and 160 *E. nitens* samples were used (Table 1); half of each species was tested in a dry condition (i.e., 80 and 80 low *MC* samples for *P. radiata* and *E. nitens,* respectively) and the rest of the samples were measured in a wet condition. Both the *P. radiata* and *E. nitens* samples were straight-grain and defect-free, and the tension parallel to the grain was tested. Samples were cut to the dimensions: 600 mm (length) × 50 mm (width) × 10 mm (thickness) with a narrow part with a cross-sectional area of 10 × 10 mm^2^ and a length of 100 mm (Figure 1a). The length and the width of the Group I samples were in the grain direction and the radial direction, respectively.

**Figure 2:**
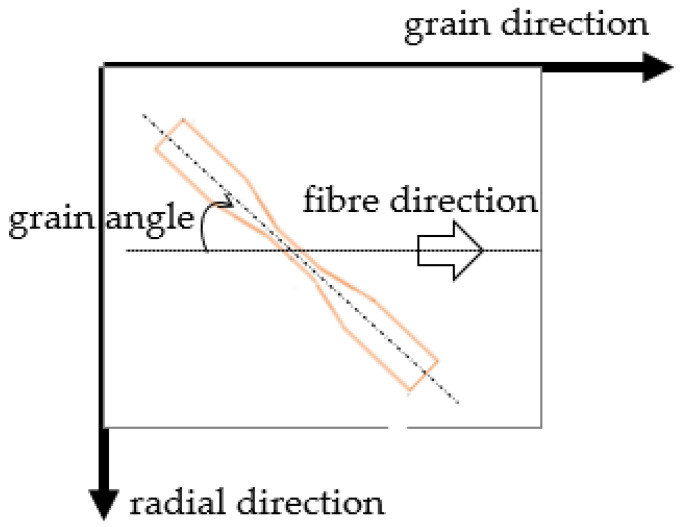
Cut method for Group II samples with grain angles of 10° and 45°.

For Group II, a total of 80 *E. nitens* samples were used to explore the anisotropic tensile behaviours of *E. nitens* timber. The cut method of Group II samples is provided in Figure 2. The grain angles of 0°, 10°, 45°, and 90°, respectively, were represented. To ensure that they have similar properties, each pair of the Group II samples (one for 0°, one for 10°, one for 45°, and one for 90°) was cut from the same board. Ten tensile samples for the grain angle of 0° in Group II were prepared in order to match the number of samples with grain angles of 10° and 45°, which were cut with the angle between the wood fibres and the longitudinal axis of the sample (Figure 2). Ten low *MC* (dry) and ten high *MC* (wet) samples were tested for each grain angle. The grain angle was measured following the direction of the fibre in the longitude of the centre part of the sample and checked after failure. Two sets of dimensions for the samples were used: the first was as for the Group I samples (Figure 1a), while the second was 600 mm (length) × 50 mm (width) × 10 mm (thickness) (Figure 1b). The first sample dimension type was waisted to ensure that failure occurred within the central part for grain angles of 0°, 10°, or 45°, while the second sample dimension type was used for testing in tension at a grain angle of 90° (i.e., perpendicular to the grain), as it is hard to manufacture waisted tensile samples at a grain angle of 90° (Figure 1b).

For Group III, the untreated *E. nitens* samples (UT-E-T0) had the same dimensions and the similar properties as the Group I dry samples. The heat-treated *E. nitens* samples (HT-E-T0) had the same dimensions as the Group I samples but were treated using the industrial thermal modification process at a maximum temperature of 220 °C for 4 h.

Just after testing, small pieces cut from the measured samples were used to determine the *MC*s and basic densities according to the work of Cheng et al. [5].

### 2.2. Testing Method and Method of Data Analysis

**Figure 3:**
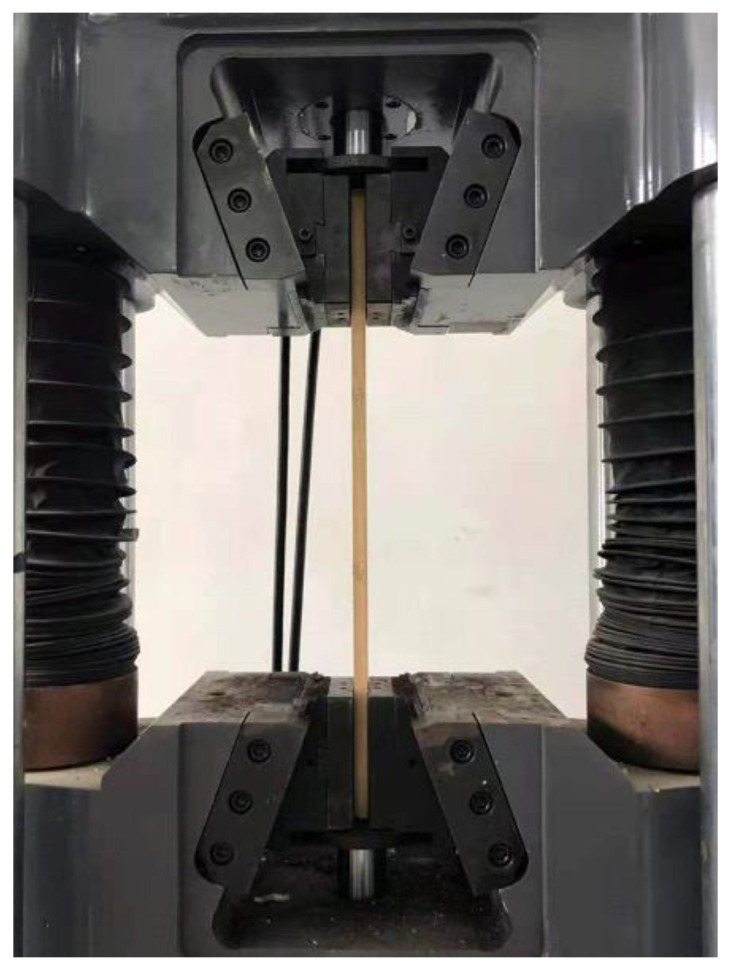
Test photo of tensile testing.

The tests were implemented on a universal testing machine (Zwick Roell-Z100, ZwickRoell LP, Kennesaw, GA, USA, capacity 100 kN) according to ASTM D143–09 [24] with relative humidity and temperatures in the range of 55–70% and 15–25 °C, respectively. The samples were carefully clamped at either end (Figure 3). Tensile testing was performed at a constant loading rate using displacement-control and discontinued when rupture failure was observed in the load-deflection curves. An increase in length over the entire sample was measured via the testing machine control signal data. Deformations were measured from the point at which tensile stress first appeared. The distance between the two grips when tension first developed was also measured as the original distance. A computer connected to a data acquisition board was used to store and analyse the data.

The tensile strain was calculated from the relative position in relation to the original distance between the two clamps. The forces were measured directly, and the stresses ($\sigma_{f})$ were calculated from the forces and the working areas (*A*) of the samples.

The tensile failure strength ($\sigma_{\alpha ,\mathrm{ten}}$) was calculated as:

(1) $$\sigma_{\alpha ,\mathrm{ten}}=\frac{f_{\mathrm{ult}}}{A}$$

in which $f_{\mathrm{ult}}$ is defined as the ultimate loading force (N), and the working area *A* (mm^2^) is the area of the failure section in the sample.

According to the stress vector relationship loaded in different grain directions, tensile strengths in L–R plane (i.e., in the plane parallel to the fibres and perpendicular to the growth rings) were calculated as:

(2) $$\sigma_{ll}=\cos^{2}\alpha \cdot \sigma_{f},\;\sigma_{rr}=\sin^{2}\alpha \cdot \sigma_{f},\;\tau_{lr}=\cos\alpha \cdot \sin\alpha \cdot \sigma_{f}$$

where $\sigma_{ll}$ and $\sigma_{rr}$ are the absolute values of the longitudinal and radial normal stresses (MPa), respectively. $\tau_{lr}$ is the absolute value of the shear stress (MPa) along the grain direction in the L–R plane. α is the grain angle, and $\sigma_{f}$ is the absolute value of the stress (MPa) in the load direction.

In this study, the moisture content (*MC*) and basic density were calculated based on AS/NZS 1080.1 [25] and the work of Cheng et al. [5], respectively. Tensile failure modes were determined directly during the test from the failed samples. The effect of a high *MC* on the failure strength of the samples was quantified by a failure strength reduction factor [5]. A failure strength reduction above the *FSP* is defined as a moisture reduction factor (*G*) to show the relative influence of moisture on failure strength above the *FSP* ($\sigma_{\alpha ,ten,MC}$) compared with that at a reference *MC* of 12% ($\sigma_{\alpha ,ten,12}$), and is given by:

(3) $$G=\frac{\sigma_{\alpha ,ten,\;MC}}{\sigma_{\alpha ,ten,12}}\;(MC>FSP)$$

where *G* is the constant depending on $\sigma_{\alpha ,ten,MC}$ and $\sigma_{\alpha ,ten,12}$*_._* The *FSP* of *E. nitens* is assumed at 30% [23].

Matlab (version R2021a, Natick, MA, USA: The MathWorks Inc.) was used to statistically analyse the experimental data. Both K–S and A–D testing [5] were used to evaluate the normal distribution of the testing data.

## 3. Results and Discussions

### 3.1. Moisture Content and Basic Densites

The basic density values of 240 *E. nitens* samples varied between 423.2 and 662.1 kg/m^3^, with a mean density value of 515.9 kg/m^3^. The basic densities of *E. nitens* in this study lie in the range of those obtained by Cheng et al. [5,10] and Derikvand et al. [7] for *E. nitens* samples, respectively, showing that the data for *E. nitens* is repeatable.

**Figure 4:**
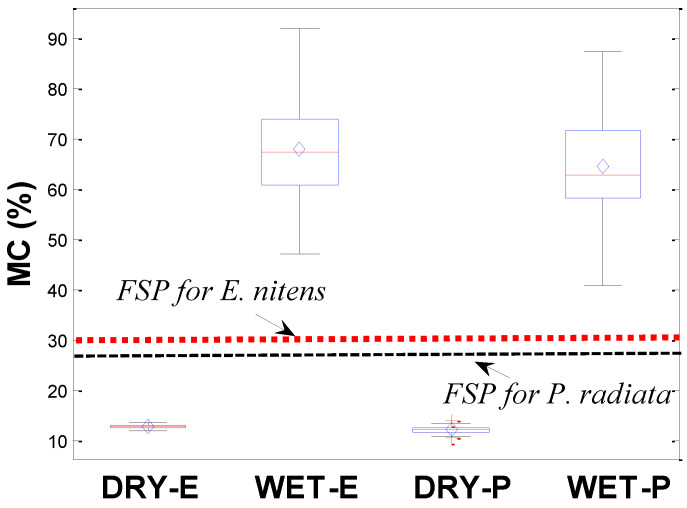
Boxplot of *MC* for *E. nitens* samples compared with the corresponding *P. radiata* samples.

Figure 4 shows a boxplot of *MC*s for *E. nitens* samples in the present study compared with the data from the corresponding *P. radiata* samples. In the present study for *E. nitens*, the *MC* varied between 11.7% and 13.5% for low *MC* samples and between 46.9% and 91.7% for high *MC* samples, with mean (COV) values of 12.7% (2.8%) and 67.7% (13.6%), respectively. *P. radiata* had a similar *MC* range to *E. nitens* samples, with the mean *MC* value of wet *P. radiata* samples being 4.8% lower than the corresponding wet *E. nitens* timber (Figure 4).

**Figure 5:**
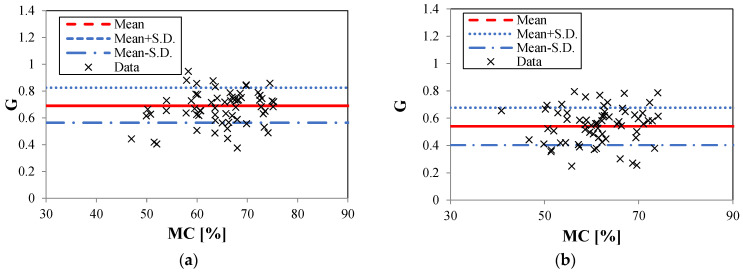
Moisture reduction factor (G) versus *MC* for Group I samples: (**a**) *E.* *nitens*; (**b**) *P. radiata.* S.D. denotes standard deviations.

Both the mean moisture contents of this research are far above the *FSP*. The Wood Handbook and previous studies have found that there are no fundamental changes in failure strength for an *MC* above the *FSP* [9,11,14]. Therefore, the 4.8% difference in *MC* does not significantly influence the values of the failure strengths studied. This indicates that the measured failure strengths from both *P. radiata* and *E. nitens* samples are comparable. Wet *P. radiata* samples were lower in failure strength compared to the corresponding *E. nitens* samples (Figure 5). This does not mean that the moisture content does not affect the failure strength of *E. nitens*. It simply means that the tensile strength of *E. nitens* is less sensitive to moisture content than that of *P. radiata*.

### 3.2. Statistical Distributions of Tensile Failure Strength Parallel to the Grain

**Figure 6:**
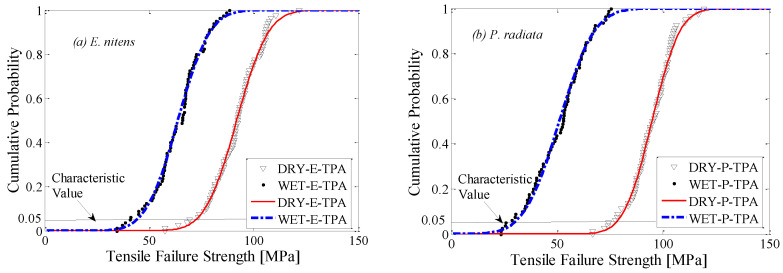
Cumulative probability distributions of experimental tensile failure strength (points) for low and high *MC* samples: (**a**) *E. nitens*; (**b**) *P. radiata*. The lines are the fitted theoretical normal distributions.

In this section, the merit of the application of *E. nitens* is explored and compared with the traditional construction material, *P. radiata*, based on the Group I tests. Figure 6 shows the empirical probability distributions of the tensile failure strengths parallel to the grain (TPA) with the fitted theoretical normal distributions. The distribution of TPA strength at a low *MC* was narrow compared to the high *MC* cases due to the high *MC* samples covering a broader *MC* range. Compared with *P. radiata,* the probability distribution of the wet *E. nitens* samples lay in a higher value range, implying that the design value of *E. nitens* was higher than *P. radiata* with a high *MC*.

Table 2 contains a statistical evaluation of the tensile failure strengths parallel to the grain (TPA) for the Group I samples. The normal probability distribution was a good fit for all data sets, which was confirmed by both Kolmogorov–Smirnov (K–S) [5,26] and Anderson–Darling (A–D) [5,27] tests (Table 2).

Table 3 presents a statistical summary of the tensile failure strength parallel to the grain for Group I samples. The basic fitted parameters for normal distributions and the characteristic values are also provided. The results revealed that the dry samples had closed mean values of failure strengths, while the mean TPA value of *P. radiata* was lower than *E. nitens* in wet conditions. Compared with *E. nitens*, the high moisture content produced a greater decline in tensile failure strength for *P. radiata.* This finding was consistent with previous research into the flexural characteristics of *E. nitens* compared with *P. radiata* [5]. This is because bending strength is related to tensile strength as the samples finally fail in tension. The 5%-quantile values of the normal distribution of *E. nitens* for low and high *MC*s were 72.4 MPa and 44.1 MPa (Table 3), respectively, which were the basis for using *E. nitens* as a potential structural tensile member. The moisture reduction factor (G) for the TPA strength of *E. nitens* was 0.69 at the mean level, which was relatively higher than those which have been used for construction timbers, for example *P. radiata* (0.53 at the mean level in this study).

This demonstrated that plantation *E. nitens* shows a great potential for use as a tensile member when exposed to water because of the lower strength reduction when the moisture content exceeds the fibre saturation point. In the following sections, further understanding of the tensile mechanical performance of plantation *E. nitens* timber with low and high *MC*s from a fibre-managed plantation resource will be discussed, based on the Group II tests.

### 3.3. Stress–Strain Curves

**Figure 7:**
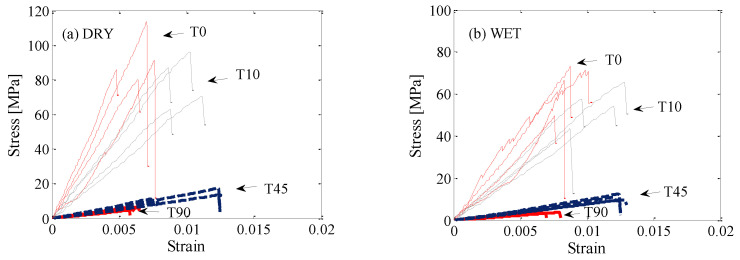
Stress–strain curves of *E. nitens* tensile samples with both low (dry) and high (wet) moisture contents for four angles α = 0°, 10°, 45°, and 90°; Left plot (**a**), dry samples; right plot (**b**), wet samples; T0, α = 0°; T10, α = 10°; T45, α = 45°; T90, α = 90°. The different lines in the same colour in each figure represent four representative replicate tests.

Figure 7 represents the tensile stress–strain curves of *E. nitens* samples in both dry and wet conditions at grain angles of 0°, 10°, 45°, and 90°. Generally, for both low (dry) and high (wet) moisture contents, the stress increased when the strain increased, and an approximately linear relationship between stress and strain was observed until the peak stress was reached. The measured stress dropped suddenly following its peak value. The peak stress was equal to the failure strength of the testing sample (Figure 7). During testing, no obvious elasticity or proportionality limit was observed; hence, the sample was failed while in the elastic range. As the grain angle increased, the slope of the stress–strain curve decreased, which demonstrated that the tensile stiffness decreased with an increase in grain angle. Not surprisingly, samples were stiffer in tension parallel to the grain than for the other grain angle loading cases, and the lowest stiffness and lowest failure strength were observed for loads perpendicular to the grain (grain angle 90°).

The linear range of stress–strain curves for the wet samples ended at somewhat lower stress values compared to the linear range of the corresponding dry samples. More ductility in the tensile behaviour was found in the wet samples (Figure 7), since moisture makes the wood fibres’ ductility increase [28]. The experimental data in the research showed that the high moisture content (wet) samples exhibited a considerably larger strain at a lower failure strength, and plus, the grain angle had a greater influence on the tensile behaviour of *E. nitens* than the moisture content.

### 3.4. Failure Modes

**Figure 8:**
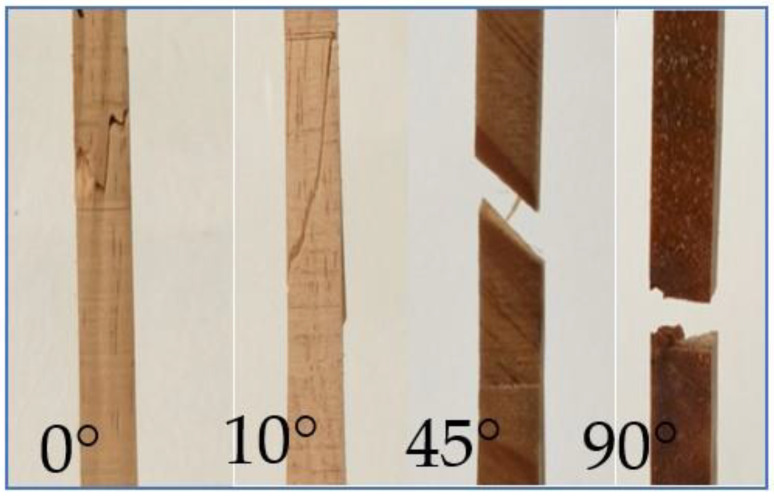
Typical failure modes of the tensile samples for four grain directions; the photo for a grain angle of 90° is from a side view.

The fibre-directed tensile failure mode was classified according to its shape [29]. The failure mode linked strongly with its grain angle, as shown in Figure 8.

A localised brittle fracture of *E. nitens* timber was found under tensile loading at a grain angle of 0° for both dry and wet conditions. For grain angles of 10° and 45°, cross-grain tensile failures in the narrow part of the samples were identified, with visible cracks extending along the grain. All the samples, both dry and wet, at a grain angle of 90° showed a clean tensile failure along the grain direction. This is because the tensile failure mode perpendicular to the grain differs from that parallel to the grain due to the wood’s microstructure [21]: cells are arranged along the wood fibre with hemicellulose connecting the fibre. This structure results in failure behaviour in tension from the fracture of the wood fibre or the breakage of the bonds between the fibres, respectively, for tension along the grain or tension perpendicular to the grain. It was found that the tensile failure mode of *E. nitens* timber depends mainly on the grain angle.

### 3.5. The Effect of Internal Stresses versus the Grain Angle

**Figure 9:**
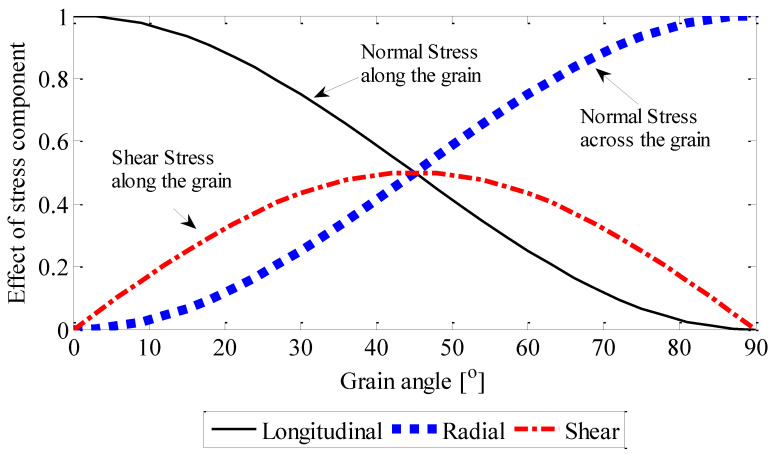
The effect of stress component ($\sigma_{ij}/\sigma_{f}$) versus grain angle. $\sigma_{ij}$ (*i*, *j* = *L*, *R*) is the stress component, and $\sigma_{f}$ is the stress in the load direction at a tensile loading of 1 kN; symbols *L* and *R* represent the principal anatomical directions: i.e., longitudinal direction and radial direction.

The above experiments examined the tensile performance of *E. nitens* with loading to different grain angles. The components of the stress variables versus the grain angle were then examined to explore the internal stress state within the *E. nitens* samples. The stress components calculated from Equation (2) at a tensile loading of 1 kN are presented in Figure 9. The tensile load value chosen here is within the capacity of wet samples loaded perpendicular to the grain. The shear stress component increased following the grain angle from 0° to 45°; then, after the shear stress component reached its peak at a grain angle of 45°, it decreased with a further increase in the grain angle. The normal stress component along the grain decreased as the grain angle increased, while the normal stress component across the grain increased as the grain angle increased. At a grain angle of 0°, the normal stress component along the grain was dominant, compared with the corresponding shear stress component and the normal stress component across the grain, which led to a localised brittle fracture of the *E. nitens* timber. At loading directions of 10° and 45°, shear stress existed and therefore, the failure mode of *E. nitens* timber was cross-grain tension failure. For a grain angle of 90°, the effect of normal stress across the grain was significant, correlating with a clean tensile failure along the grain direction due to a breakage of the bonds between fibres. Thus, the internal stress state within *E. nitens* timber links strongly with its failure mode. This is why the tensile failure of *E. nitens* timber is significantly influenced by the grain angle compared with moisture content.

### 3.6. Failure Envelopes and Failure Strengths

**Figure 10:**
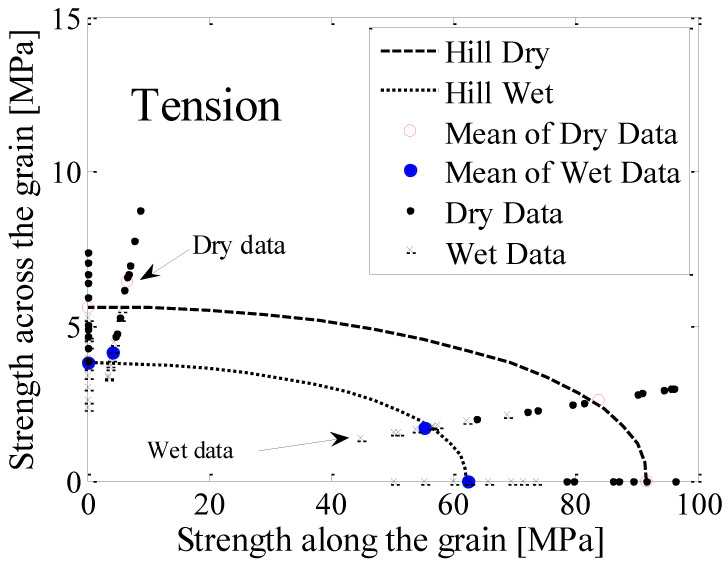
The tensile failure envelopes in the longitudinal and radial planes for *E. nitens* timber in dry (*MC* = 12%) and wet (*MC* > *FSP*) states compared with the mean values of experimental data (the circles). The failure envelopes were determined based on Hill criteria [30] using the values of the parameters presented in Table 4.

The failure envelopes are examined here for the further study of establishing stress limits for structural sizing based on the envelope failure curves [30]. The Hill failure criterion [30] was adopted as it is widely used in timber engineering [30,31]. For both low (dry) and high (wet) *MC*s, the failure envelopes of *E. nitens* in tension, calculated according to the equations given in Table 4, were 1/4 elliptical arcs which expanded as the moisture content decreased (Figure 10).

Since the failure strength perpendicular to the grain was much lower than that parallel to the grain, the long axis of the elliptical arc coincided with the grain direction. This implied that the tensile strength of *E. nitens* timber was obviously dependent on the grain angle, and the anisotropic tensile behaviour of *E. nitens* timber both below and above the *FSP* was proved. The correlation between the experimental results and the Hill criteria confirmed this finding.

Next, the tensile failure strengths in this study were compared with the findings from compression testing of *E. nitens* samples with low and high *MC*s [10]. The compressive result was obtained from a previous study on *E. nitens* samples [10]. Table 5 shows the mean failure strengths of *E. nitens* timber at grain angles of 0°, 45°, and 90° with both low (dry) and high (wet) *MC*s. For both tension and compression, as the grain angle increased, the failure strength decreased. Compared with the dry samples, lower failure strengths were found for wet samples for the three grain angle situations (0°, 45°, and 90°), implying an increase in the risk of *E. nitens* failure with an increase in *MC*.

A significant difference in the value of failure strengths of *E. nitens* in tension and compression was found (Table 5). Consistent with previous findings on other species [11], along the grain, a higher tensile failure strength was found than that under compression both above and below the *FSP*, i.e., the compressive strength of the wood was almost two times lower than the tensile strength. This agreed with previous studies on dry wood, which showed that wood is an anisotropic material with different strengths in tension and compression [29,32]. This anisotropy is due to the cellular nature of wood and its growth. The wood cell wall contains cellulose, hemicelluloses, and lignin, which interact to form a natural composite material [21]. Because long and narrow cells act as columns of wood fibres in the grain direction and the hemicellulose bonds the wood fibres as fibril networks, wood has different post-failure mechanisms for tension and compression and loads parallel to the grain or perpendicular to the grain [32]. Tension strength parallel to the grain (TPA) is suggested for use with *E. nitens* timber of low and high *MC*s in practical applications, as this exploits the highest strength.

The present work extends this research to wet (*MC* > *FSP*) samples and considers the failure strength with loading to different grain directions. The present study found that the failure strengths and the mechanical behaviour of *E. nitens* timber in tension and compression were also different above the *FSP*. Compared with the data from the testing of thermally-modified *E. nitens* timber, a similar reduction in the mean strength level was found (0.75 for heat-treated/untreated Group III samples, and 0.69 for wet/dry Group I *E nitens* samples). This is because a high moisture affects the wood cellulose polymers and reduces hydrogen bonding between the polymers [18]. The effect of the high moisture content on the tensile characterisation of *E. nitens* timber is reversible. However, the degradation of thermally-modified *E. nitens* timber is irreversible due to the thermal modification process which contributes to the degradation of hemicellulose [33,34] and changes the components of the wood natural composite [11,18,35].

## 4. Conclusions

This study characterised the anisotropic tensile preformation of *Eucalyptus nitens* (*E. nitens)* timber above the *FSP*, which had not previously been studied and quantified. This was also the first study to examine the suitability of *Eucalyptus nitens* timber, both below and above the *FSP*, as a structural material loaded in tension. Tensile experiments were carried out on 240 *E. nitens* samples using a universal testing machine. The anisotropic behaviour of *E. nitens* was tested at various load-to-grain angles, as well as at low and high *MC*s.

The findings can be summarised as follows:

- When *MC* > *FSP*, the traditional structural timber, *P. radiata*, showed a greater decrease in longitudinal tensile failure strength than was found for *E. nitens*. The mean strength reduction of *E. nitens* was less than that of traditional construction timber, for example *P. radiata*, indicating the *E. nitens* shows promise for use in the building industry, especially when exposed to water. The design characteristic values of *E. nitens* timber were 72.4 MPa and 44.1 Mpa, respectively.
- Samples with both low and high *MC*s failed by brittle fracture, while the high *MC* samples achieved lower values of tensile strength. The tensile failure strengths were demonstrated to be moisture content sensitive. The different failure strengths of *E. nitens* wood in tension and compression were also found both below and above the *FSP.* A similar reduction in the strength was found for high moisture content samples compared with thermally-modified *E. nitens* timber, indicating that moisture content affects *E. nitens* wood cellulose polymers.
- Plantation *E. nitens* timber in tension was highly anisotropic with respect to grain angle, and its ductility increased as *MC* increased. Compared with moisture content, the grain angle had a stronger effect on the tensile failure of *E. nitens* timber due to the micro-structure of *E. nitens* timber and differences in the internal stress state. At grain angles of 0°, the normal stress along the grain was dominant compared with the corresponding shear stress and normal stress across the grain, leading to simple tensile failure. At a loading direction of 45°, shear stresses played an important role, resulting in cross-grain tensile failure. For a grain angle of 90°, the effect of normal stress across the grain was significant, and therefore bonds between fibres in the micro-structure broke and clean tensile failure along the grain direction was observed.

This study could provide a basis for future research to assess the vulnerability of *E. nitens* tensile members for structural applications.

## Figures and Tables

**Table 1:** Summary of experiments.

	Species	Moisture Content (*MC*)	Code	Number of Replicated Samples	Grain Angle
GROUP I	*E. nitens*	12%	DRY-E-TPA	80	0°
		>30%	WET-E-TPA	80	0°
	*P. radiata*	12%	DRY-P-TPA	80	0°
		>30%	WET-P-TPA	80	0°
GROUP II	*E. nitens*	12%	DRY-E-T0	10	0°
			DRY-E-T10	10	10°
			DRY-E-T45	10	45°
			DRY-E-T90	10	90°
		>30%	WET-E-T0	10	0°
			WET-E-T10	10	10°
			WET-E-T45	10	45°
			WET-E-T90	10	90°
GROUP III	Untreated *E. nitens* (kiln dried)	12%	UT-E-T0	10	0°
	Heat-treated *E. nitens*		HT-E-T0	10	0°

**Table 2:** Goodness-of-fit for normal distribution of the longitudinal tensile failure strength.

		Kolmogorov–Smirnov (K–S) Test			Anderson–Darling (A–D) Test		
		Maximum Absolute Difference	Critical Value	Acceptance	Squared Distance	Critical Value	Acceptance
*E. nitens*	DRY-E-TPA	0.0716	0.1496	Yes	0.31	0.54	Yes
	WET-E-TPA	0.0897	0.1496	Yes	0.34	0.47	Yes
*P. radiata*	DRY-P-TPA	0.0605	0.1496	Yes	0.34	0.49	Yes
	WET-P-TPA	0.0792	0.1496	Yes	0.35	0.44	Yes

**Table 3:** Tensile failure strength parallel to the grain (TPA).

Tensile Failure Strength	*E. nitens*		*P. radiata*	
	*MC* = 12%	*MC* > *FSP*	*MC* = 12%	*MC* > *FSP*
Mean (Mpa)	91.9	63.8	94.7	51.1
Coefficients of variations	13.0%	18.8%	11.0%	25.4%
Characteristic value, *f_n_* (Mpa)	72.4	44.1	77.6	29.8

**Table 4:** Hill criteria showing in Figure 10 below.

Code	Formulae	Notes
Hill Dry	$\frac{\sigma_{l}^{2}}{{\left(91.6\right)}_{}^{2}}+\frac{\sigma_{r}^{2}}{{\left(5.6\right)}_{}^{2}}-\frac{\sigma_{l}\cdot \sigma_{r}}{{\left(91.6\right)}_{}^{2}}+\frac{\tau_{lr}^{2}}{{\left(12.9\right)}_{}^{2}}$ = 1	Hill criterion for dry samples in tension
Hill Wet	$\frac{\sigma_{l}^{2}}{{\left(62.1\right)}_{}^{2}}+\frac{\sigma_{r}^{2}}{{\left(3.8\right)}_{}^{2}}-\frac{\sigma_{l}\cdot \sigma_{r}}{{\left(62.1\right)}_{}^{2}}+\frac{\tau_{lr}^{2}}{{\left(8.3\right)}_{}^{2}}$ = 1	Hill criterion for wet samples in tension

**Table 5:** The failure strengths of *E. nitens* timber in both dry (*MC* = 12%) and wet (*MC* = 66% > *FSP*) conditions at grain angles of 0°, 45°, and 90°.

**Grain Angle (°)**			**Compression [10]**			**Tension**		
			0	45	90	0	45	90
Failure strength (MPa)	DRY (*MC* = 12%)	Mean	43.2	18.0	8.3	91.6	12.9	5.6
		S.D.*	5.5	2.4	2.0	10.9	2.4	1.2
		N **	10	10	10	10	10	10
	WET (*MC* > *FSP*)	Mean	23.9	9.6	4.6	62.1	8.3	3.8
		S.D. *	3.1	1.8	1.0	7.9	1.4	0.9
		N **	10	10	10	10	10	10

* S.D. denotes standard deviations; ** N is the number of the replicate tests.

## Data Availability

Not applicable.
